# A case report of pediatric systemic lupus erythematosus with diffuse alveolar hemorrhage following COVID-19 infection: Causation, association, or chance?

**DOI:** 10.1097/MD.0000000000030071

**Published:** 2022-08-19

**Authors:** Ali Alsuheel Asseri, Raneem Al-Murayeh, Abdoh M. Abudiah, Elsayed I. Elgebally, Abdullah M. Aljaser

**Affiliations:** a Department of Child Health, College of Medicine, King Khalid University, Abha, Saudi Arabia; b College of Medicine, King Khalid University, Abha, Saudi Arabia; c Department of Pediatrics, Abha Maternity and Children Hospital, Abha, Saudi Arabia; d Department of Pediatrics, Saudi German Hospital, Aseer, Saudi Arabia; e Department of Pediatrics, Menoufia University, Shebeen Al-Kom, Egypt.

**Keywords:** autoimmune diseases, children, COVID-19, diffuse alveolar hemorrhage, systemic lupus erythematosus

## Abstract

**Rationale:**

Diffuse alveolar hemorrhage (DAH) is a rare manifestation of childhood systemic lupus erythematosus (SLE) that can be life-threatening. Several reports have linked previous or concurrent coronavirus disease (COVID-19) infections with a high prevalence of autoimmune and autoinflammatory disorders.

**Patient concerns:**

We report a case of a 13-year-old female who presented with DAH due to SLE 2 months after a laboratory-confirmed severe COVID-19 infection.

**Diagnoses:**

The patient was diagnosed with DAH due to SLE 2 months after a laboratory-confirmed severe COVID-19 infection.

**Interventions and outcomes:**

The patient was treated with intravenous methylprednisolone pulse, broad-spectrum antibiotics, and supportive measures. In addition, she received 6 sessions of plasma exchange and maintenance methylprednisolone therapy (2 mg/kg/day). The patient then improved and was discharged on prednisolone, hydroxychloroquine, and azathioprine.

**Lessons:**

We suggest plasmapheresis be considered a treatment for SLE-associated DAH in the context of active disease when conventional treatment has failed to induce a rapid response. In addition, further studies are needed to assess the role of COVID-19 as an autoimmune disease trigger, particularly for SLE.

## 1. Introduction

Systemic lupus erythematosus (SLE) is a chronic relapsing and remitting autoimmune disease characterized by multiorgan involvement and variable disease severity. The exact incidence and prevalence of childhood SLE is unknown; however, several reports have estimated a prevalence of 3.3 to 8.8 per 100,000 children.^[1,2]^ SLE is characterized by the production of autoantibodies that frequently affect the skin, joints, kidneys, and the nervous, hematologic, and cardiovascular systems.^[2–4]^ Diffuse alveolar hemorrhage (DAH) is a rare manifestation of childhood SLE that can be life-threatening; therefore, timely intervention is critical.^[5]^ The exact etiology of SLE is unknown, but it is considered a complex disease incorporating genetic, hormonal, immunologic, and environmental factors.^[6]^ Previous studies have reported that some viruses may be implicated as potential triggers or pathogenic agents of autoimmune conditions, such as SLE.^[6–9]^ Epstein-Barr virus (EBV), parvovirus, cytomegalovirus (CMV), and severe acute respiratory syndrome coronavirus 2 (SARS-CoV-2) have all been frequently reported in patients with SLE pathogenesis with different proposed mechanisms.^[6,8–10]^ One potential mechanism is molecular mimicry, in which immune responses to viral antigens shift against self-antigens, leading to autoimmunity.^[6,7,11]^

Coronavirus disease 2019 (COVID-19) is a viral respiratory infection caused by an emerging novel coronavirus, SARS-CoV-2, which is frequently associated with respiratory failure, pneumonia, and acute respiratory distress syndrome.^[12,13]^ Since the beginning of the COVID-19 pandemic, several reports have linked previous or concurrent COVID-19 infections to a high prevalence of autoimmune and autoinflammatory disorders.^[9,14–16]^ Recently, an adult SLE case report has been reported following severe COVID-19 infection.^[9]^ In addition, Gracia-Ramos et al^[17]^ recently published a comprehensive review of the emergence of autoimmune diseases during the COVID-19 pandemic, concluding that some autoimmune diseases are associated with COVID-19 infection. However, they recommended further investigation to further elucidate this association.^[17]^

SLE involvement in the respiratory system is reported in approximately half of adult patients and includes pleuritis, interstitial pneumonitis, pulmonary hypertension, and DAH; however, these disease manifestations are very rare in children.^[2,18,19]^ DAH is a rare but life-threatening complication of SLE, with high mortality and long-term pulmonary morbidities. Due to immune complex-induced injury to the microvasculature, the hemorrhage originates in the interstitial capillaries and alveoli and leads to a sudden drop in hemoglobin. Hemoptysis, bilateral chest X-ray (CXR) opacifications, and abrupt hypoxemic respiratory failure are the most common manifestations.^[20,21]^ We herein report a case of a 13-year-old female who presented with DAH due to SLE, 2 months after a laboratory-confirmed, severe COVID-19 infection.

## 2. Case presentation

A 13-year-old female with an otherwise unremarkable history presented to the local hospital during the COVID-19 pandemic with fever, cough, and shortness of breath that had lasted for 2 days. There was history of contact with a household with confirmed COVID-19. The symptoms included a runny nose and sore throat. Upon arrival, she had a high temperature (38.5°C), a respiratory rate of 28 breaths/min, a heart rate of 122/min, a blood pressure of 115/70 mm Hg, and an oxygen saturation of 72% on ambient air. The initial examination revealed that the patient was well-nourished and had severe respiratory distress and pallor. Chest examination revealed bilaterally decreased air entry with inspiratory crackles.

The laboratory findings were as follows: hemoglobin (Hb) level: 12 g/dL (reference range [RR], 11.5–15.5 g/dL), mean corpuscular volume: 69.4 fL (RR, 77.0–95.0 fL), mean corpuscular hemoglobin level: 19.6 pg (RR, 25.0–33.0 pg), and hematocrit: 36. Her white blood cell and platelet counts were within normal limits. Her serum lactate dehydrogenase level was elevated to 887 international unit (IU)/L (RR, 125–243 IU/L), C-reactive protein level was 34 mg/L (<10 mg/L), and the erythrocyte sedimentation rate was 74 mm/hr (0–20 mm/hr). Her serum ferritin level was 550 ng/mL (RR, 9–185 ng/mL), and her D-dimer was 65108 ng/mL (RR, <500). Her electrolytes, as well as kidney and liver function, were normal. The COVID-19 screening with nasopharyngeal polymerase chain reaction was positive. CXR revealed a multifocal air-space disease consistent with COVID-19 pneumonia. The patient was admitted and treated for severe COVID-19 pneumonia. She was started on broad-spectrum antibiotics, favipiravir, systemic steroids, a zinc supplement, and supportive measures (hydration and oxygen). After 5 days in the hospital, she was discharged in good condition (normal work of breathing, oxygen saturation, and hemoglobin level).

**Figure 1.**
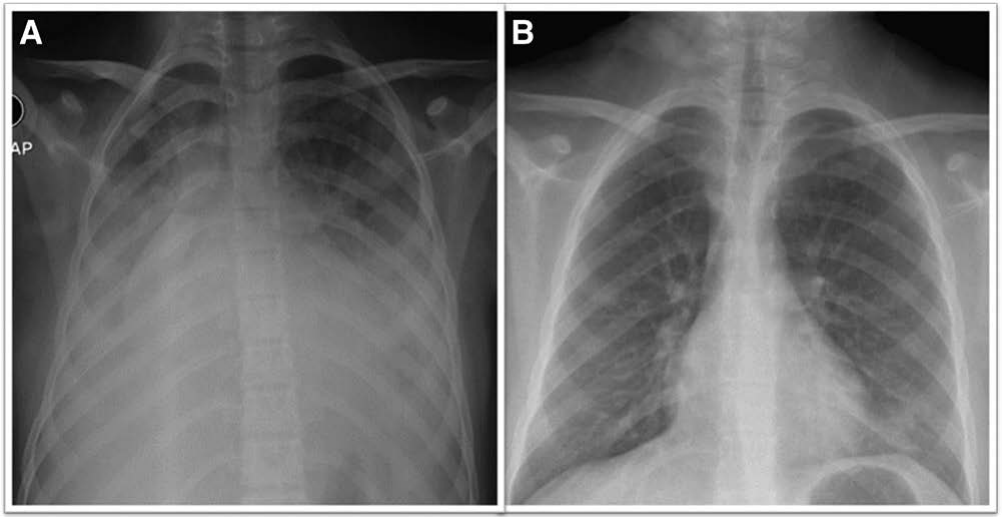
(A) Frontal CXR showing significant bilateral consolidation silhouetting bilateral hemidiaphragm and cardiac borders. (B) Follow-up CXR after 2 mo, showing significant improvement with minimal residual patchy infiltration in the left lower lobe. AP = Anteroposterior, CXR = chest X-ray.

**Figure 2.**
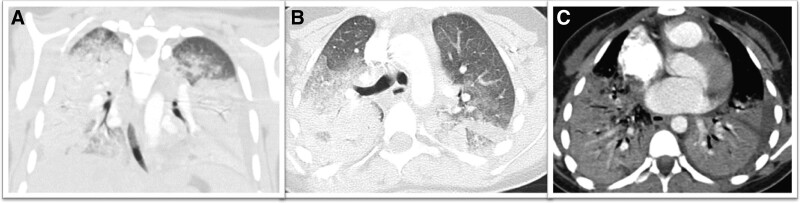
Chest CT images of the patient. (A) Coronal reformat CT lung showing bilateral and diffuse infiltrates with air bronchogram. (B) Axial chest CT window showing significant bilateral consolidation and ground-glass appearance (right more than left). (C) Enhanced axial chest CT mediastinal window showing significant bibasilar airspace disease. CT = computed tomography.

Two months after the first episode, she was admitted to Abha Maternity and Children’s Hospital with anemia and acute respiratory failure. She was admitted into the intensive care unit, and a high-flow nasal cannula was started to maintain oxygenation. CXR showed significant bilateral consolidation silhouetting bilateral hemidiaphragm and cardiac borders (Fig. 1A). Chest computed tomography showed significant bilateral consolidation and ground-glass appearance (Fig. 2). A direct Coombs test yielded negative results, whereas an indirect Coombs test yielded positive results. Urine analysis showed 4+ proteinuria and 3+ blood. Detailed laboratory data are shown in Table 1. The patient was positive for anti-SARS-CoV-2 immunoglobulin M and immunoglobulin G (IgG) antibodies, but tests for all other antiviral antibodies were negative, including EBV, parvovirus B19, CMV, retroviruses, and human immunodeficiency virus. Transthoracic echocardiography showed a normal heart without any evidence of pulmonary hypertension or pericardial effusion. Because the severe respiratory insufficiency, acute anemia, and bilateral lung opacities suggested alveolar hemorrhages, flexible bronchoscopy was performed. Bronchoalveolar lavage of the right middle lobe showed blood-stained fluid and yielded abundant hemosiderin-laden macrophages. The patient was treated with intravenous methylprednisolone pulse, broad-spectrum antibiotics, and supportive measures. An autoantibody panel was positive for antinuclear antibody (ANA) and anti-double-stranded deoxyribonucleic acid (anti-ds DNA). Additionally, complement levels were examined, revealing reduced C3 and C4 levels. Positivity for ANA and anti-ds DNA indicated an autoimmune etiology. Based on these results, the Pediatric Rheumatology team was consulted, and another autoimmune panel was ordered for the patient, which was negative for antiglomerular basement membrane antibodies, rheumatoid factors, antineutrophil cytoplasmic antibodies, proteinase-3 antibodies, anti-cardiolipin IgG antibodies, beta-2 glycoprotein antibodies, and anti-Smith antibodies. These findings supported a diagnosis of DAH due to SLE.

**Table 1:** Laboratory data.

Variables	Reference range	Value
Blood and biochemical tests		
White blood cells (×10^9^/L)	4500–11,000	9.44
Absolute lymphocyte count, per mm^3^	1000–4800	690
Absolute neutrophil count, per mm^3^	1800–7700	8600
Hemoglobin (g/dL)	12.0–16.0	8.61
Hematocrit	36%–46%	27.4%
Platelets (×10^9^/L)	150,000–450,000	326
CRP (mg/dL)	<0.30	10
ESR (mm/hr)	0–13	84
Ferritin, µg/L	30–300	343
Procalcitonin, µg/L	0.00–0.08	0.45
Troponin, ng/mL	<2.0	1.4
D-dimer (μg/L)	<500	470
BUN (mg/dL)	8.0–25	13
Creatinine (mg/dL)	0.30–1.00	0.5
Sodium (mEq/L)	135–145	139
ALT (IU/L)	10–55	34
AST (IU/L)	9.0–32	31
Albumin (g/dL)	3.4–5.4	1.8
LDH (IU/L)	13–60	736
ANA (CU)	0–20	>200 CU
C3 mg/dL	90–180	32.9
C4 mg/dL	90–180	32.9
Anti-dsDNA	0–27	1245.9 IU/mL
c-ANCA, CU	Negative < 20	<2.3
p-ANCA	Negative < 20	<3.2
Anti-cardiolipin IgM/IgG, CU	0–20	2.7/14.2
Cardiolipin IgG, CU	Negative < 20	6.7
Cardiolipin IgM, CU	Negative < 20	6.3
HIV I and II Ag and Ab	Nonreactive	Nonreactive
Anti-EBV IgM antibodies	Negative	Negative
SARS-CoV-2 (IgM and IgG) antibodies	Positive	Positive

Ab = antibody, Ag = antigen, ALT = alanine transaminase, ANA = antinuclear antibody, Anti-dsDNA = Anti-double stranded DNA, AST = aspartate aminotransferase, BUN = blood urea nitrogen, C3 = complement component 3, C4 = complement component 4, c-ANCA = centrally Accentuated Antineutrophil Cytoplasmic Antibody Test, CRP = C-reactive protein, CU = chemiluminescence units, EBV = Epstein-Barr virus, ESR = erythrocyte sedimentation rate, HIV = human immunodeficiency virus, IgG = immunoglobulin G, IgM = immunoglobulin M, IU = international unit; LDH = lactate dehydrogenase, p-ANCA = perinuclear antineutrophil cytoplasmic antibody, SARS-CoV-2 = severe acute respiratory syndrome coronavirus 2.

Despite receiving pulse steroid and supportive measures, the patient did not improve. Therefore, the treating team started plasma exchange sessions. The patient received 6 sessions of plasma exchange and maintenance methylprednisolone therapy (2 mg/kg/d). Eight days after admission, the patient improved with normal work of breathing and a saturation of 95% on room air. Her hemoglobin level was 10.5 g/dL, and her CXR showed significant improvement. The patient was discharged on prednisolone, hydroxychloroquine, and azathioprine. Upon follow-up, the patient’s respiratory status had improved, evidenced by the absence of respiratory symptoms and the significant improvement in her CXR (Fig. 1B).

## 3. Discussion

We present a case of a pediatric patient with newly diagnosed SLE manifested as life-threatening DAH preceded by a severe COVID-19 infection. To the best of the authors’ knowledge, this is the first reported case of SLE triggered by COVID-19 in Saudi Arabia, although other pediatric and adult cases have been reported with different presenting syndromes.^[9,22,23]^ This case adds more complexity to the association between COVID-19 infection and the evolution of autoimmune disorders. The current case was diagnosed based on the evidence of the American College of Rheumatology criteria for SLE diagnosis, which included hematological manifestations (lymphopenia and anemia), positive ANA, positive anti-ds DNA, and renal involvement (persistent proteinuria).^[24]^ Hemoptysis, a rapid drop in the Hb level, hypoxemic respiratory failure, and diffuse CXR infiltrates are the most common manifestations of DAH.^[5,18,19]^ The current patient presented with life-threatening DAH following severe COVID-19 infection, which is considered a rare presenting complication of SLE.^[20,21]^ Several publications have reported autoinflammatory and autoimmune illnesses in patients who have recovered from COVID-19.^[14,16,17,25]^

The exact etiology and pathogenesis of SLE are unclear and may be related to genetic predisposition, autoimmunity, and viral infections.^[6]^ Infection is one of the triggers for autoimmune diseases.^[11,26]^ The association between viral infection and autoimmune disorders is complex and could trigger, worsen, or resemble SLE.^[27]^ Zamani et al^[9]^ recently reported a 38-year-old man who presented with COVID-19 infection and developed SLE 2 months later. Furthermore, several viruses such as parvovirus B19 and EBV could resemble and trigger lupus.^[11]^ Our patient tested positive for SARS-CoV-2 immunoglobulin M and IgG antibodies and was negative for other viruses (EBV, CMV, and human immunodeficiency virus). It is well known that SARS-CoV-2 and other viruses cause a cytokine storm with the overproduction of several inflammatory cytokines, such as interferon gamma, tumor necrosis factor-α, macrophage inflammatory protein-1 alpha, interleukin (IL)-1), IL-2, IL-6, and IL-7.^[8–11]^ Furthermore, a recent review by Gao et al. evaluated the association between viral infections and autoimmune illnesses, concluding that the levels of autoantibodies increased in COVID-19-infected patients, increasing the risk of autoimmune disorders.^[28]^ The presence of SARS-CoV-2 antibodies and the absence of antibodies for other viruses indicate a link between COVID-19 and SLE. However, further studies are needed to clearly identify the role of COVID-19 in the pathogenesis of SLE. In addition, we recommend long-term follow-up for patients who had severe COVID-19-induced acute respiratory distress syndrome until more data are available to confirm the link between autoimmune diseases and COVID-19 infection.

The current patient had very high levels of autoantibodies with life-threatening DAH that mandated critical care and aggressive therapies, including pulse steroid and plasmapheresis. Regardless of whether it is caused by SLE, DAH is a life-threatening condition that necessitates early treatment and a thorough investigation to identify the underlying cause.^[21]^ In a recently published systematic review, Jiang et al^[29]^ concluded that older age at DAH diagnosis, increased SLE disease duration, requirement for plasmapheresis or mechanical ventilation, and concurrent infection are risk factors associated with poor survival in SLE-related DAH. However, due to its rarity in the pediatric age group, most of the risk factors and the therapeutic interventions for that age group are extrapolated from reports on adults. The exact cause of DAH in SLE patients is unknown; however, the general view is that immune complex-induced pulmonary capillaritis or bland hemorrhage leads to damage to basement membranes and the leakage of erythrocytes into the alveolar space.^[20]^ The patient recovered after receiving steroids and plasmapheresis with complete clinical and radiological resolution of DAH.

## 4. Conclusion

We report a pediatric patient who presented with life-threatening DAH due to SLE that occurred after severe COVID-19 infection. The SLE diagnosis was based on the clinical symptoms and the presence of autoantibodies. As the DAH did not respond to the pulse steroid therapy, plasmapheresis was initiated. We suggest plasmapheresis be considered a treatment for SLE-associated DAH in the context of active disease when conventional treatment has failed to induce a rapid response. In addition, further studies are needed to assess the role of COVID-19 as an autoimmune disease trigger, particularly for SLE.

## Acknowledgments

The authors would like to thank the patient’s family. Special thanks to Dr. Mohammed Algathradi, Pediatric Radiologist, Department of Radiology, King Khalid University, Saudi Arabia, who read and reviewed the radiological pictures.

## Author contributions

Ali Asseri and Raneem Al-Murayeh contributed to the acquisition of patient information and article writing. Abdoh M. Abudiah, Elsayed I. Elgebally, and Abdullah M. Aljaser participated in the diagnosis and treatment of the patient. All authors read and approved the final article.
